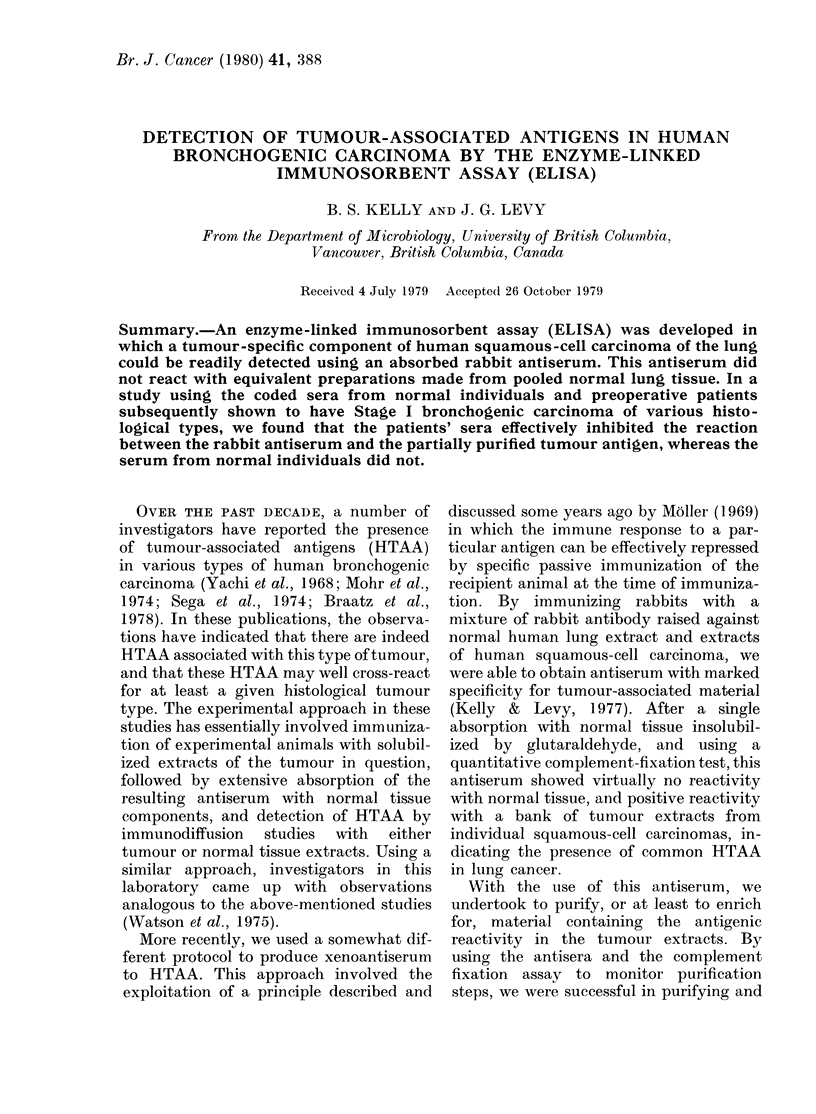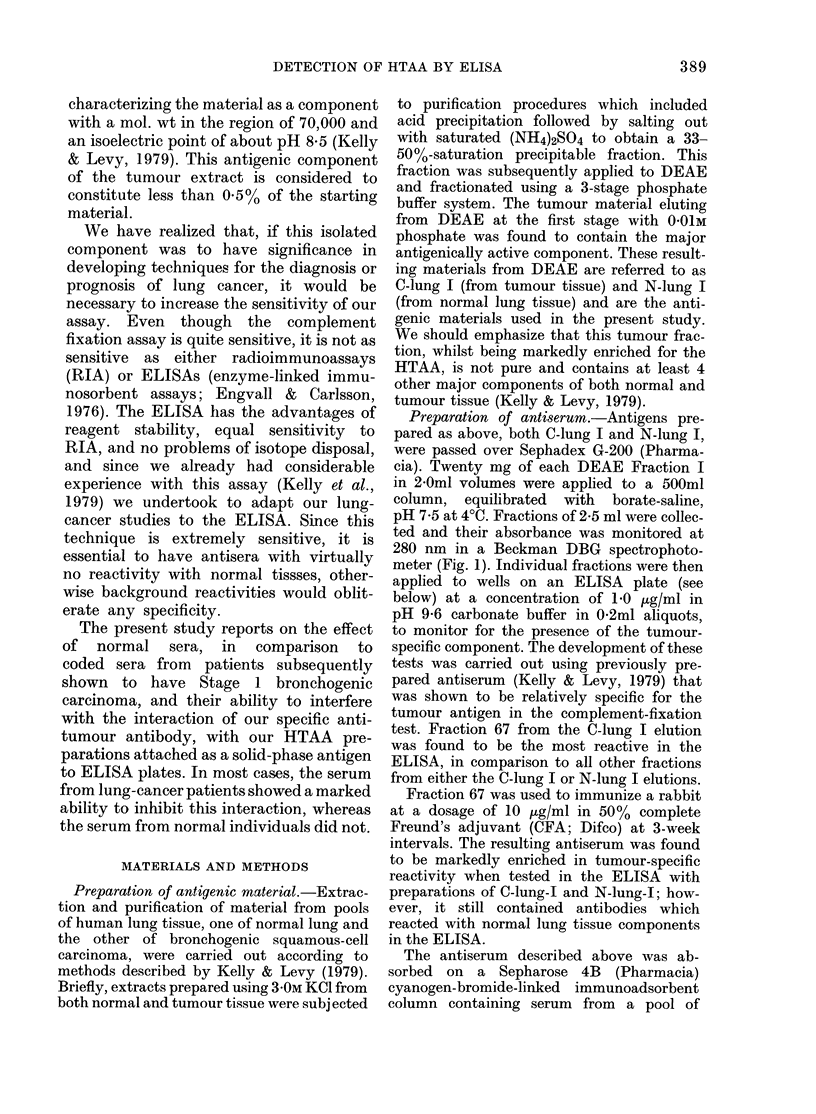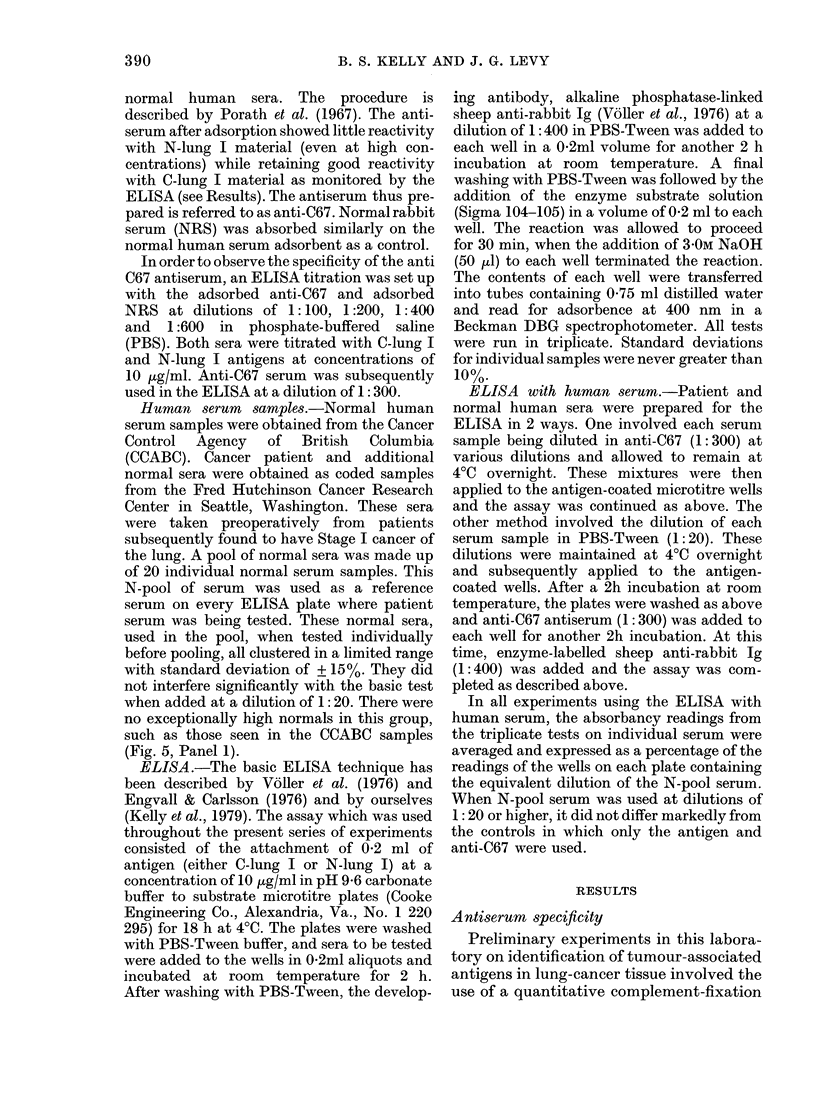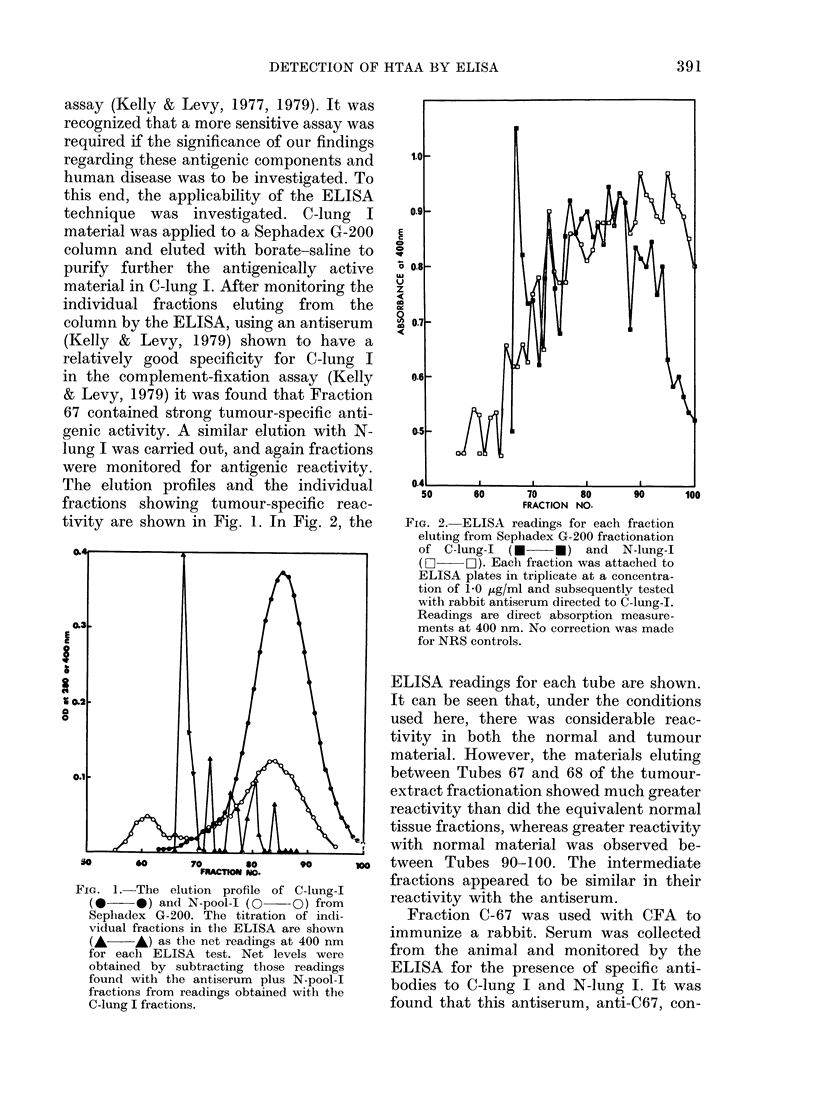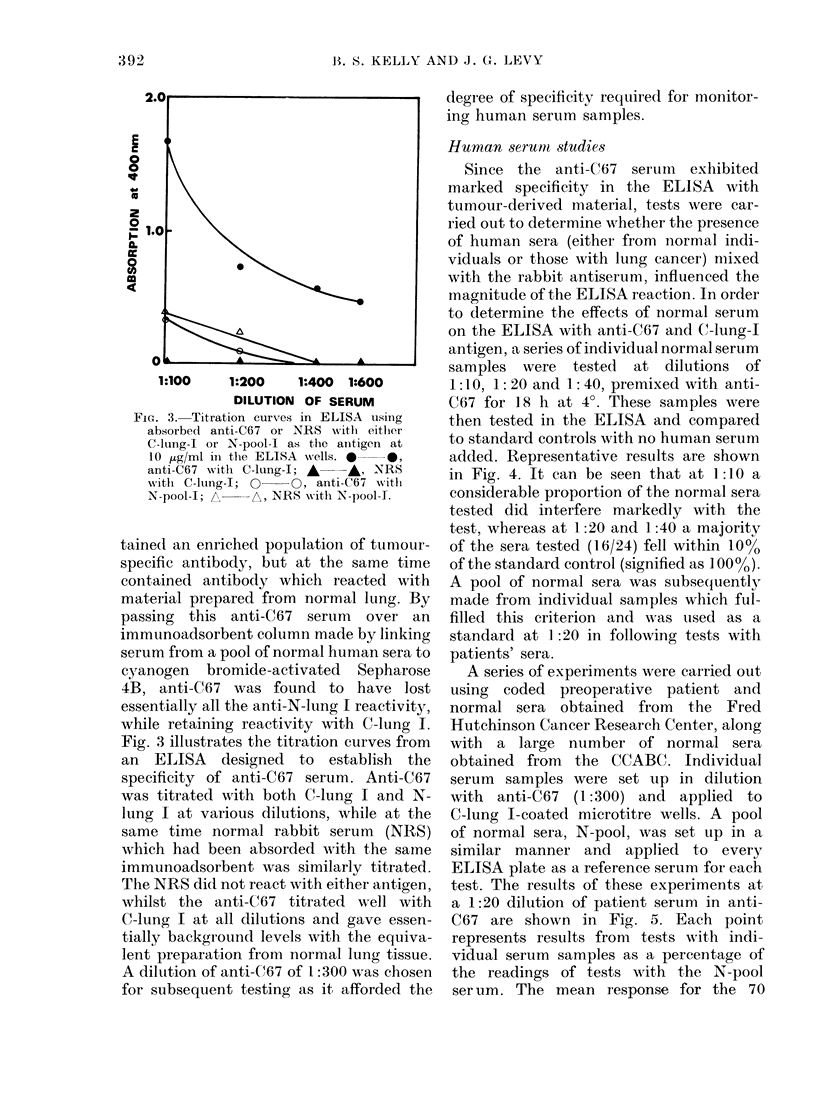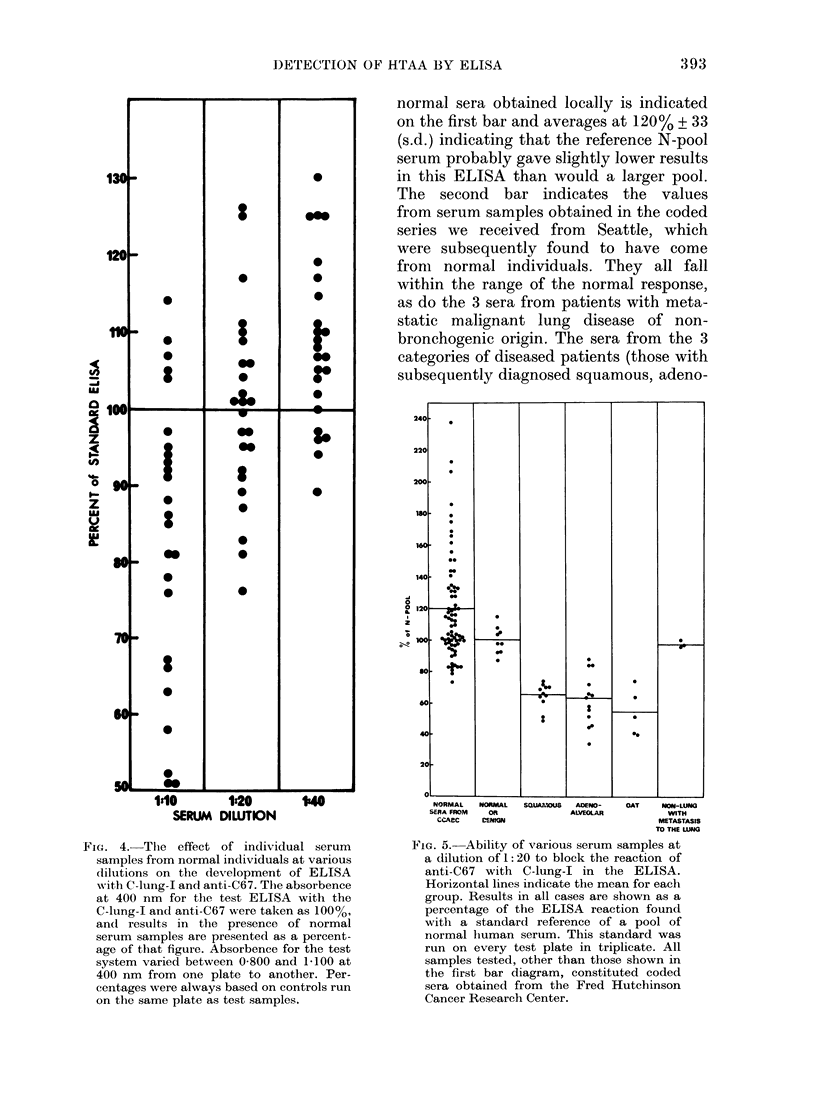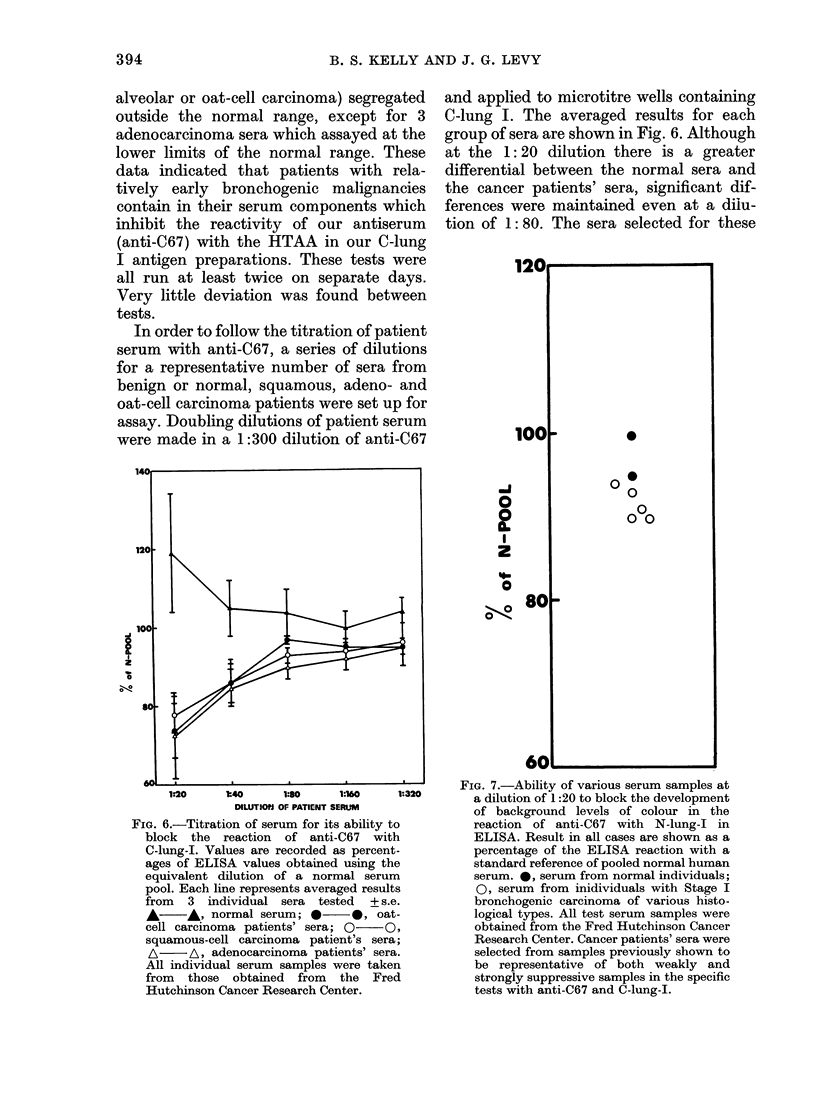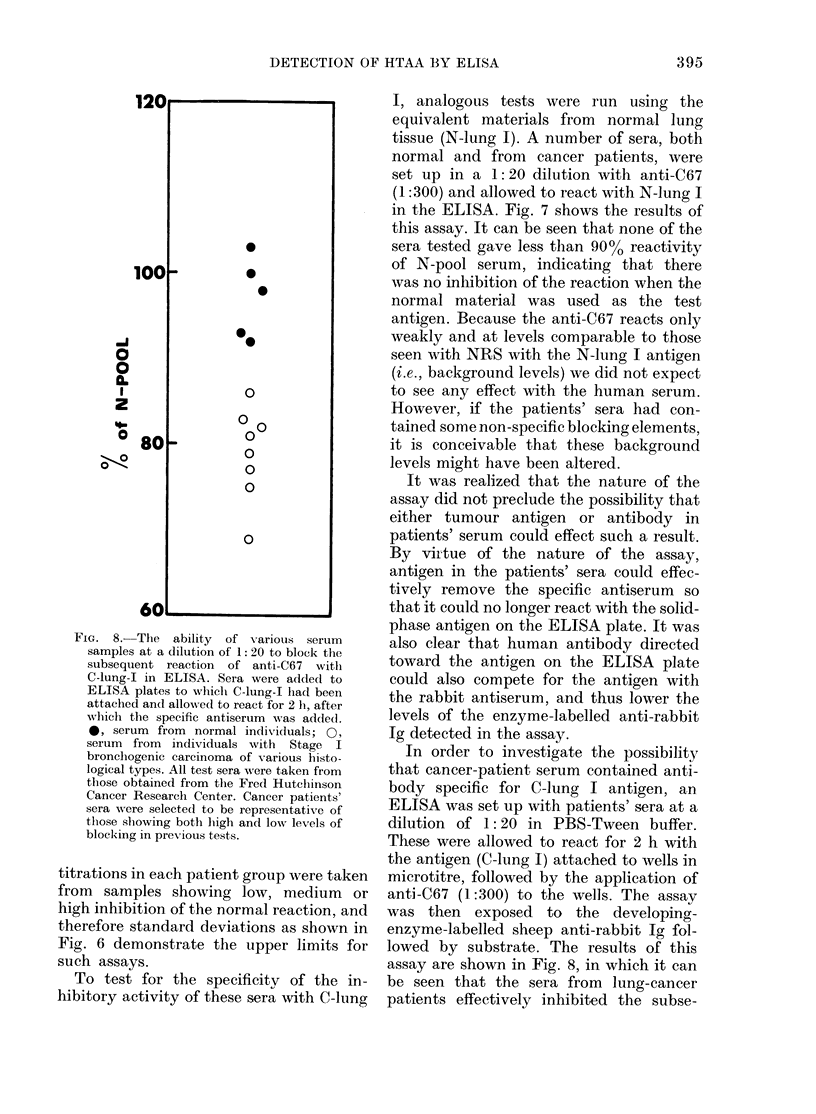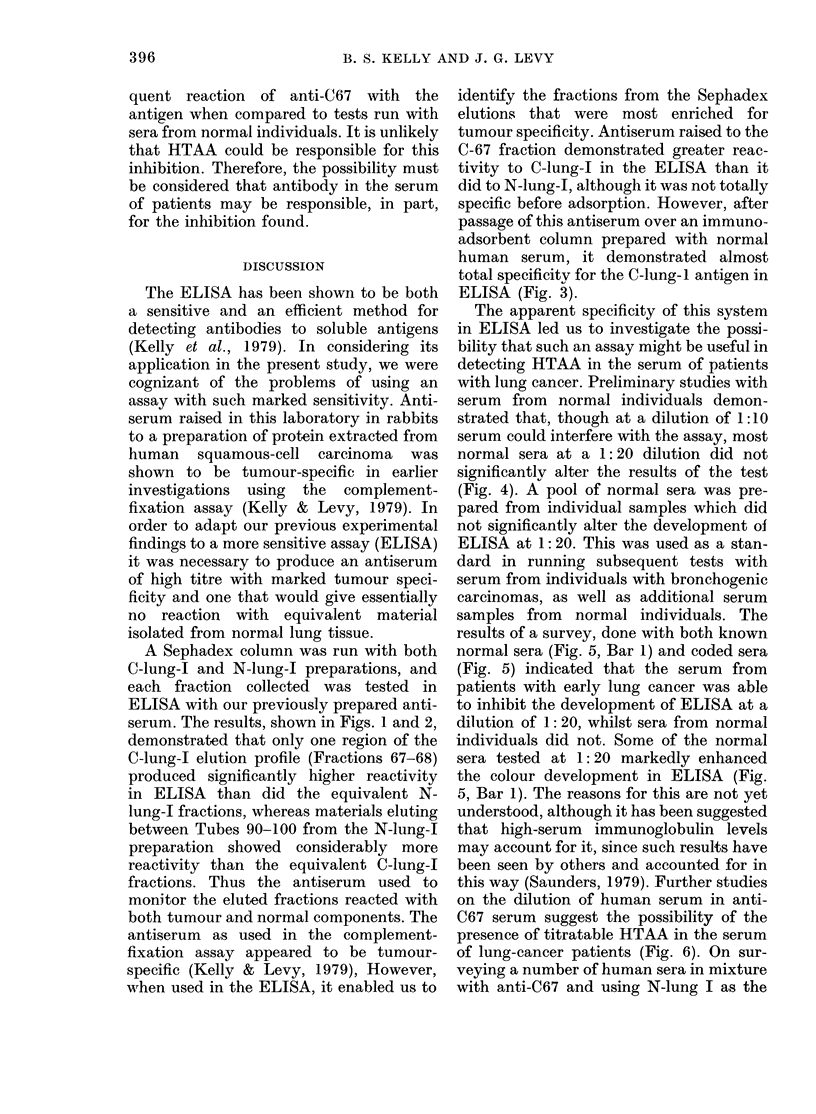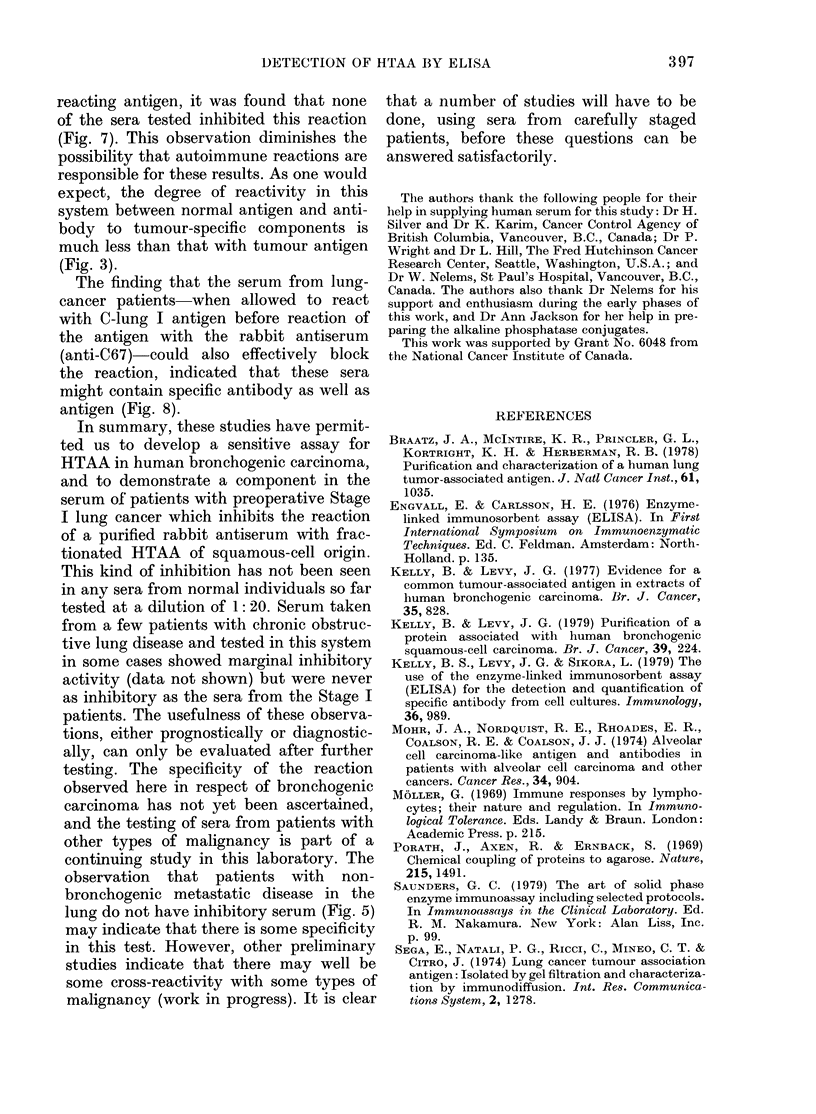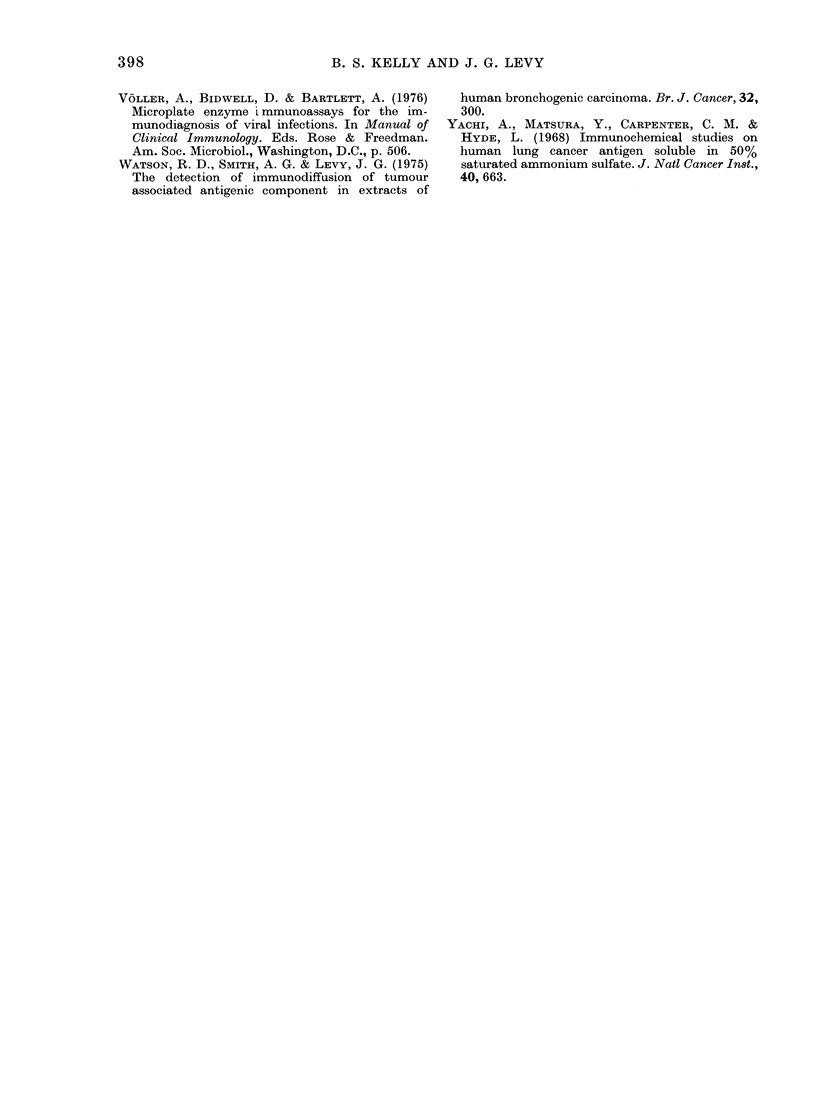# Detection of tumour-associated antigens in human bronchogenic carcinoma by the enzyme-linked immunosorbent assay (ELISA).

**DOI:** 10.1038/bjc.1980.62

**Published:** 1980-03

**Authors:** B. S. Kelly, J. G. Levy

## Abstract

An enzyme-linked immunosorbent assay (ELISA) was developed in which a tumour-specific component of human squamous-cell carcinoma of the lung could be readily detected using an absorbed rabbit antiserum. This antiserum did not react with equivalent preparations made from pooled normal lung tissue. In a study using the coded sera from normal individuals and preoperative patients subsequently shown to have Stage I bronchogenic carcinoma of various histological types, we found that the patients' sera effectively inhibited the reaction between the rabbit antiserum and the partially purified tumour antigen, whereas the serum from normal individuals did not.


*Br. J. Cancer* (1980) **41**, 388

### DETECTION OF TUMOUR-ASSOCIATED ANTIGENS IN HUMAN BRONCHOGENIC CARCINOMA BY THE ENZYME-LINKED IMMUNOSORBENT ASSAY (ELISA)

B. S. KELLY AND J. G. LEVY

*From the Department of Microbiology, University of British Columbia, Vancouver, British Columbia, Canada*

Received 4 July 1979 Accepted 26 October 1979

**Summary.—An enzyme-linked immunosorbent assay (ELISA) was developed in which a tumour-specific component of human squamous-cell carcinoma of the lung could be readily detected using an absorbed rabbit antiserum. This antiserum did not react with equivalent preparations made from pooled normal lung tissue. In a study using the coded sera from normal individuals and preoperative patients subsequently shown to have Stage I bronchogenic carcinoma of various histological types, we found that the patients' sera effectively inhibited the reaction between the rabbit antiserum and the partially purified tumour antigen, whereas the serum from normal individuals did not.**

Over the past decade, a number of investigators have reported the presence of tumour-associated antigens (HTAA) in various types of human bronchogenic carcinoma (Yachi *et al.*, 1968; Mohr *et al.*, 1974; Sega *et al.*, 1974; Braatz *et al.*, 1978). In these publications, the observations have indicated that there are indeed HTAA associated with this type of tumour, and that these HTAA may well cross-react for at least a given histological tumour type. The experimental approach in these studies has essentially involved immunization of experimental animals with solubilized extracts of the tumour in question, followed by extensive absorption of the resulting antiserum with normal tissue components, and detection of HTAA by immunodiffusion studies with either tumour or normal tissue extracts. Using a similar approach, investigators in this laboratory came up with observations analogous to the above-mentioned studies (Watson *et al.*, 1975).

More recently, we used a somewhat different protocol to produce xenoantiserum to HTAA. This approach involved the exploitation of a principle described and discussed some years ago by Möller (1969) in which the immune response to a particular antigen can be effectively repressed by specific passive immunization of the recipient animal at the time of immunization. By immunizing rabbits with a mixture of rabbit antibody raised against normal human lung extract and extracts of human squamous-cell carcinoma, we were able to obtain antiserum with marked specificity for tumour-associated material (Kelly & Levy, 1977). After a single absorption with normal tissue insolubilized by glutaraldehyde, and using a quantitative complement-fixation test, this antiserum showed virtually no reactivity with normal tissue, and positive reactivity with a bank of tumour extracts from individual squamous-cell carcinomas, indicating the presence of common HTAA in lung cancer.

With the use of this antiserum, we undertook to purify, or at least to enrich for, material containing the antigenic reactivity in the tumour extracts. By using the antisera and the complement fixation assay to monitor purification steps, we were successful in purifying and characterizing the material as a component with a mol. wt in the region of 70,000 and an isoelectric point of about pH 8·5 (Kelly & Levy, 1979). This antigenic component of the tumour extract is considered to constitute less than 0·5% of the starting material.

We have realized that, if this isolated component was to have significance in developing techniques for the diagnosis or prognosis of lung cancer, it would be necessary to increase the sensitivity of our assay. Even though the complement fixation assay is quite sensitive, it is not as sensitive as either radioimmunoassays (RIA) or ELISAs (enzyme-linked immunosorbent assays; Engvall & Carlsson, 1976). The ELISA has the advantages of reagent stability, equal sensitivity to RIA, and no problems of isotope disposal, and since we already had considerable experience with this assay (Kelly *et al.*, 1979) we undertook to adapt our lung-cancer studies to the ELISA. Since this technique is extremely sensitive, it is essential to have antisera with virtually no reactivity with normal tissses, otherwise background reactivities would obliterate any specificity.

The present study reports on the effect of normal sera, in comparison to coded sera from patients subsequently shown to have Stage 1 bronchogenic carcinoma, and their ability to interfere with the interaction of our specific anti-tumour antibody, with our HTAA preparations attached as a solid-phase antigen to ELISA plates. In most cases, the serum from lung-cancer patients showed a marked ability to inhibit this interaction, whereas the serum from normal individuals did not.

### MATERIALS AND METHODS

*Preparation of antigenic material.*—Extraction and purification of material from pools of human lung tissue, one of normal lung and the other of bronchogenic squamous-cell carcinoma, were carried out according to methods described by Kelly & Levy (1979). Briefly, extracts prepared using 3·0M KCl from both normal and tumour tissue were subjected to purification procedures which included acid precipitation followed by salting out with saturated $(NH_4)_2SO_4$ to obtain a 33–50%-saturation precipitable fraction. This fraction was subsequently applied to DEAE and fractionated using a 3-stage phosphate buffer system. The tumour material eluting from DEAE at the first stage with 0·01M phosphate was found to contain the major antigenically active component. These resulting materials from DEAE are referred to as C-lung I (from tumour tissue) and N-lung I (from normal lung tissue) and are the antigenic materials used in the present study. We should emphasize that this tumour fraction, whilst being markedly enriched for the HTAA, is not pure and contains at least 4 other major components of both normal and tumour tissue (Kelly & Levy, 1979).

*Preparation of antiserum.*—Antigens prepared as above, both C-lung I and N-lung I, were passed over Sephadex G-200 (Pharmacia). Twenty mg of each DEAE Fraction I in 2·0ml volumes were applied to a 500ml column, equilibrated with borate-saline, pH 7·5 at 4°C. Fractions of 2·5 ml were collected and their absorbance was monitored at 280 nm in a Beckman DBG spectrophotometer (Fig. 1). Individual fractions were then applied to wells on an ELISA plate (see below) at a concentration of 1·0 μg/ml in pH 9·6 carbonate buffer in 0·2ml aliquots, to monitor for the presence of the tumour-specific component. The development of these tests was carried out using previously prepared antiserum (Kelly & Levy, 1979) that was shown to be relatively specific for the tumour antigen in the complement-fixation test. Fraction 67 from the C-lung I elution was found to be the most reactive in the ELISA, in comparison to all other fractions from either the C-lung I or N-lung I elutions.

Fraction 67 was used to immunize a rabbit at a dosage of 10 μg/ml in 50% complete Freund's adjuvant (CFA; Difco) at 3-week intervals. The resulting antiserum was found to be markedly enriched in tumour-specific reactivity when tested in the ELISA with preparations of C-lung-I and N-lung-I; however, it still contained antibodies which reacted with normal lung tissue components in the ELISA.

The antiserum described above was absorbed on a Sepharose 4B (Pharmacia) cyanogen-bromide-linked immunoadsorbent column containing serum from a pool of normal human sera. The procedure is described by Porath *et al.* (1967). The antiserum after adsorption showed little reactivity with N-lung I material (even at high concentrations) while retaining good reactivity with C-lung I material as monitored by the ELISA (see Results). The antiserum thus prepared is referred to as anti-C67. Normal rabbit serum (NRS) was absorbed similarly on the normal human serum adsorbent as a control.

In order to observe the specificity of the anti C67 antiserum, an ELISA titration was set up with the adsorbed anti-C67 and adsorbed NRS at dilutions of 1:100, 1:200, 1:400 and 1:600 in phosphate-buffered saline (PBS). Both sera were titrated with C-lung I and N-lung I antigens at concentrations of 10 $\mu$g/ml. Anti-C67 serum was subsequently used in the ELISA at a dilution of 1:300.

*Human serum samples.*—Normal human serum samples were obtained from the Cancer Control Agency of British Columbia (CCABC). Cancer patient and additional normal sera were obtained as coded samples from the Fred Hutchinson Cancer Research Center in Seattle, Washington. These sera were taken preoperatively from patients subsequently found to have Stage I cancer of the lung. A pool of normal sera was made up of 20 individual normal serum samples. This N-pool of serum was used as a reference serum on every ELISA plate where patient serum was being tested. These normal sera, used in the pool, when tested individually before pooling, all clustered in a limited range with standard deviation of $\pm 15\%$. They did not interfere significantly with the basic test when added at a dilution of 1:20. There were no exceptionally high normals in this group, such as those seen in the CCABC samples (Fig. 5, Panel 1).

*ELISA.*—The basic ELISA technique has been described by Völler *et al.* (1976) and Engvall & Carlsson (1976) and by ourselves (Kelly *et al.*, 1979). The assay which was used throughout the present series of experiments consisted of the attachment of 0·2 ml of antigen (either C-lung I or N-lung I) at a concentration of 10 $\mu$g/ml in pH 9·6 carbonate buffer to substrate microtitre plates (Cooke Engineering Co., Alexandria, Va., No. 1 220 295) for 18 h at 4°C. The plates were washed with PBS-Tween buffer, and sera to be tested were added to the wells in 0·2ml aliquots and incubated at room temperature for 2 h. After washing with PBS-Tween, the developing antibody, alkaline phosphatase-linked sheep anti-rabbit Ig (Völler *et al.*, 1976) at a dilution of 1:400 in PBS-Tween was added to each well in a 0·2ml volume for another 2 h incubation at room temperature. A final washing with PBS-Tween was followed by the addition of the enzyme substrate solution (Sigma 104–105) in a volume of 0·2 ml to each well. The reaction was allowed to proceed for 30 min, when the addition of 3·0M NaOH (50 $\mu$l) to each well terminated the reaction. The contents of each well were transferred into tubes containing 0·75 ml distilled water and read for adsorbence at 400 nm in a Beckman DBG spectrophotometer. All tests were run in triplicate. Standard deviations for individual samples were never greater than 10%.

*ELISA with human serum.*—Patient and normal human sera were prepared for the ELISA in 2 ways. One involved each serum sample being diluted in anti-C67 (1:300) at various dilutions and allowed to remain at 4°C overnight. These mixtures were then applied to the antigen-coated microtitre wells and the assay was continued as above. The other method involved the dilution of each serum sample in PBS-Tween (1:20). These dilutions were maintained at 4°C overnight and subsequently applied to the antigen-coated wells. After a 2h incubation at room temperature, the plates were washed as above and anti-C67 antiserum (1:300) was added to each well for another 2h incubation. At this time, enzyme-labelled sheep anti-rabbit Ig (1:400) was added and the assay was completed as described above.

In all experiments using the ELISA with human serum, the absorbancy readings from the triplicate tests on individual serum were averaged and expressed as a percentage of the readings of the wells on each plate containing the equivalent dilution of the N-pool serum. When N-pool serum was used at dilutions of 1:20 or higher, it did not differ markedly from the controls in which only the antigen and anti-C67 were used.

### RESULTS

### *Antiserum specificity*

Preliminary experiments in this laboratory on identification of tumour-associated antigens in lung-cancer tissue involved the use of a quantitative complement-fixation assay (Kelly & Levy, 1977, 1979). It was recognized that a more sensitive assay was required if the significance of our findings regarding these antigenic components and human disease was to be investigated. To this end, the applicability of the ELISA technique was investigated. C-lung I material was applied to a Sephadex G-200 column and eluted with borate–saline to purify further the antigenically active material in C-lung I. After monitoring the individual fractions eluting from the column by the ELISA, using an antiserum (Kelly & Levy, 1979) shown to have a relatively good specificity for C-lung I in the complement-fixation assay (Kelly & Levy, 1979) it was found that Fraction 67 contained strong tumour-specific antigenic activity. A similar elution with N-lung I was carried out, and again fractions were monitored for antigenic reactivity. The elution profiles and the individual fractions showing tumour-specific reactivity are shown in Fig. 1. In Fig. 2, the

Fig. 1.—The elution profile of C-lung-I (●——●) and N-pool-I (○——○) from Sephadex G-200. The titration of individual fractions in the ELISA are shown (▲——▲) as the net readings at 400 nm for each ELISA test. Net levels were obtained by subtracting those readings found with the antiserum plus N-pool-I fractions from readings obtained with the C-lung I fractions.

Fig. 2.—ELISA readings for each fraction eluting from Sephadex G-200 fractionation of C-lung-I (■——■) and N-lung-I (□——□). Each fraction was attached to ELISA plates in triplicate at a concentration of 1·0 μg/ml and subsequently tested with rabbit antiserum directed to C-lung-I. Readings are direct absorption measurements at 400 nm. No correction was made for NRS controls.

ELISA readings for each tube are shown. It can be seen that, under the conditions used here, there was considerable reactivity in both the normal and tumour material. However, the materials eluting between Tubes 67 and 68 of the tumour-extract fractionation showed much greater reactivity than did the equivalent normal tissue fractions, whereas greater reactivity with normal material was observed between Tubes 90–100. The intermediate fractions appeared to be similar in their reactivity with the antiserum.

Fraction C-67 was used with CFA to immunize a rabbit. Serum was collected from the animal and monitored by the ELISA for the presence of specific antibodies to C-lung I and N-lung I. It was found that this antiserum, anti-C67, con-

FIG. 3.—Titration curves in ELISA using absorbed anti-C67 or NRS with either C-lung-I or N-pool-I as the antigen at 10 μg/ml in the ELISA wells. ●——●, anti-C67 with C-lung-I; ▲——▲, NRS with C-lung-I; ○——○, anti-C67 with N-pool-I; △——△, NRS with N-pool-I.

tained an enriched population of tumour-specific antibody, but at the same time contained antibody which reacted with material prepared from normal lung. By passing this anti-C67 serum over an immunoadsorbent column made by linking serum from a pool of normal human sera to cyanogen bromide-activated Sepharose 4B, anti-C67 was found to have lost essentially all the anti-N-lung I reactivity, while retaining reactivity with C-lung I. Fig. 3 illustrates the titration curves from an ELISA designed to establish the specificity of anti-C67 serum. Anti-C67 was titrated with both C-lung I and N-lung I at various dilutions, while at the same time normal rabbit serum (NRS) which had been absorded with the same immunoadsorbent was similarly titrated. The NRS did not react with either antigen, whilst the anti-C67 titrated well with C-lung I at all dilutions and gave essentially background levels with the equivalent preparation from normal lung tissue. A dilution of anti-C67 of 1:300 was chosen for subsequent testing as it afforded the degree of specificity required for monitoring human serum samples.

*Human serum studies*

Since the anti-C67 serum exhibited marked specificity in the ELISA with tumour-derived material, tests were carried out to determine whether the presence of human sera (either from normal individuals or those with lung cancer) mixed with the rabbit antiserum, influenced the magnitude of the ELISA reaction. In order to determine the effects of normal serum on the ELISA with anti-C67 and C-lung-I antigen, a series of individual normal serum samples were tested at dilutions of 1:10, 1:20 and 1:40, premixed with anti-C67 for 18 h at 4°. These samples were then tested in the ELISA and compared to standard controls with no human serum added. Representative results are shown in Fig. 4. It can be seen that at 1:10 a considerable proportion of the normal sera tested did interfere markedly with the test, whereas at 1:20 and 1:40 a majority of the sera tested (16/24) fell within 10% of the standard control (signified as 100%). A pool of normal sera was subsequently made from individual samples which fulfilled this criterion and was used as a standard at 1:20 in following tests with patients' sera.

A series of experiments were carried out using coded preoperative patient and normal sera obtained from the Fred Hutchinson Cancer Research Center, along with a large number of normal sera obtained from the CCABC. Individual serum samples were set up in dilution with anti-C67 (1:300) and applied to C-lung I-coated microtitre wells. A pool of normal sera, N-pool, was set up in a similar manner and applied to every ELISA plate as a reference serum for each test. The results of these experiments at a 1:20 dilution of patient serum in anti-C67 are shown in Fig. 5. Each point represents results from tests with individual serum samples as a percentage of the readings of tests with the N-pool serum. The mean response for the 70

FIG. 4.—The effect of individual serum samples from normal individuals at various dilutions on the development of ELISA with C-lung-I and anti-C67. The absorbence at 400 nm for the test ELISA with the C-lung-I and anti-C67 were taken as 100%, and results in the presence of normal serum samples are presented as a percentage of that figure. Absorbence for the test system varied between 0·800 and 1·100 at 400 nm from one plate to another. Percentages were always based on controls run on the same plate as test samples.

normal sera obtained locally is indicated on the first bar and averages at 120% ± 33 (s.d.) indicating that the reference N-pool serum probably gave slightly lower results in this ELISA than would a larger pool. The second bar indicates the values from serum samples obtained in the coded series we received from Seattle, which were subsequently found to have come from normal individuals. They all fall within the range of the normal response, as do the 3 sera from patients with metastatic malignant lung disease of non-bronchogenic origin. The sera from the 3 categories of diseased patients (those with subsequently diagnosed squamous, adeno-

FIG. 5.—Ability of various serum samples at a dilution of 1:20 to block the reaction of anti-C67 with C-lung-I in the ELISA. Horizontal lines indicate the mean for each group. Results in all cases are shown as a percentage of the ELISA reaction found with a standard reference of a pool of normal human serum. This standard was run on every test plate in triplicate. All samples tested, other than those shown in the first bar diagram, constituted coded sera obtained from the Fred Hutchinson Cancer Research Center.

alveolar or oat-cell carcinoma) segregated outside the normal range, except for 3 adenocarcinoma sera which assayed at the lower limits of the normal range. These data indicated that patients with relatively early bronchogenic malignancies contain in their serum components which inhibit the reactivity of our antiserum (anti-C67) with the HTAA in our C-lung I antigen preparations. These tests were all run at least twice on separate days. Very little deviation was found between tests.

In order to follow the titration of patient serum with anti-C67, a series of dilutions for a representative number of sera from benign or normal, squamous, adeno- and oat-cell carcinoma patients were set up for assay. Doubling dilutions of patient serum were made in a 1:300 dilution of anti-C67 and applied to microtitre wells containing C-lung I. The averaged results for each group of sera are shown in Fig. 6. Although at the 1:20 dilution there is a greater differential between the normal sera and the cancer patients' sera, significant differences were maintained even at a dilution of 1:80. The sera selected for these

FIG. 6.—Titration of serum for its ability to block the reaction of anti-C67 with C-lung-I. Values are recorded as percentages of ELISA values obtained using the equivalent dilution of a normal serum pool. Each line represents averaged results from 3 individual sera tested ±s.e. ▲——▲, normal serum; ●——●, oat-cell carcinoma patients' sera; ○——○, squamous-cell carcinoma patient's sera; △——△, adenocarcinoma patients' sera. All individual serum samples were taken from those obtained from the Fred Hutchinson Cancer Research Center.

FIG. 7.—Ability of various serum samples at a dilution of 1:20 to block the development of background levels of colour in the reaction of anti-C67 with N-lung-I in ELISA. Result in all cases are shown as a percentage of the ELISA reaction with a standard reference of pooled normal human serum. ●, serum from normal individuals; ○, serum from inidividuals with Stage I bronchogenic carcinoma of various histological types. All test serum samples were obtained from the Fred Hutchinson Cancer Research Center. Cancer patients' sera were selected from samples previously shown to be representative of both weakly and strongly suppressive samples in the specific tests with anti-C67 and C-lung-I.

FIG. 8.—The ability of various serum samples at a dilution of 1:20 to block the subsequent reaction of anti-C67 with C-lung-I in ELISA. Sera were added to ELISA plates to which C-lung-I had been attached and allowed to react for 2 h, after which the specific antiserum was added. ●, serum from normal individuals; ○, serum from individuals with Stage I bronchogenic carcinoma of various histological types. All test sera were taken from those obtained from the Fred Hutchinson Cancer Research Center. Cancer patients' sera were selected to be representative of those showing both high and low levels of blocking in previous tests.

titrations in each patient group were taken from samples showing low, medium or high inhibition of the normal reaction, and therefore standard deviations as shown in Fig. 6 demonstrate the upper limits for such assays.

To test for the specificity of the inhibitory activity of these sera with C-lung I, analogous tests were run using the equivalent materials from normal lung tissue (N-lung I). A number of sera, both normal and from cancer patients, were set up in a 1:20 dilution with anti-C67 (1:300) and allowed to react with N-lung I in the ELISA. Fig. 7 shows the results of this assay. It can be seen that none of the sera tested gave less than 90% reactivity of N-pool serum, indicating that there was no inhibition of the reaction when the normal material was used as the test antigen. Because the anti-C67 reacts only weakly and at levels comparable to those seen with NRS with the N-lung I antigen (*i.e.*, background levels) we did not expect to see any effect with the human serum. However, if the patients' sera had contained some non-specific blocking elements, it is conceivable that these background levels might have been altered.

It was realized that the nature of the assay did not preclude the possibility that either tumour antigen or antibody in patients' serum could effect such a result. By virtue of the nature of the assay, antigen in the patients' sera could effectively remove the specific antiserum so that it could no longer react with the solid-phase antigen on the ELISA plate. It was also clear that human antibody directed toward the antigen on the ELISA plate could also compete for the antigen with the rabbit antiserum, and thus lower the levels of the enzyme-labelled anti-rabbit Ig detected in the assay.

In order to investigate the possibility that cancer-patient serum contained antibody specific for C-lung I antigen, an ELISA was set up with patients' sera at a dilution of 1:20 in PBS-Tween buffer. These were allowed to react for 2 h with the antigen (C-lung I) attached to wells in microtitre, followed by the application of anti-C67 (1:300) to the wells. The assay was then exposed to the developing-enzyme-labelled sheep anti-rabbit Ig followed by substrate. The results of this assay are shown in Fig. 8, in which it can be seen that the sera from lung-cancer patients effectively inhibited the subsequent reaction of anti-C67 with the antigen when compared to tests run with sera from normal individuals. It is unlikely that HTAA could be responsible for this inhibition. Therefore, the possibility must be considered that antibody in the serum of patients may be responsible, in part, for the inhibition found.

### DISCUSSION

The ELISA has been shown to be both a sensitive and an efficient method for detecting antibodies to soluble antigens (Kelly *et al.*, 1979). In considering its application in the present study, we were cognizant of the problems of using an assay with such marked sensitivity. Antiserum raised in this laboratory in rabbits to a preparation of protein extracted from human squamous-cell carcinoma was shown to be tumour-specific in earlier investigations using the complement-fixation assay (Kelly & Levy, 1979). In order to adapt our previous experimental findings to a more sensitive assay (ELISA) it was necessary to produce an antiserum of high titre with marked tumour specificity and one that would give essentially no reaction with equivalent material isolated from normal lung tissue.

A Sephadex column was run with both C-lung-I and N-lung-I preparations, and each fraction collected was tested in ELISA with our previously prepared antiserum. The results, shown in Figs. 1 and 2, demonstrated that only one region of the C-lung-I elution profile (Fractions 67–68) produced significantly higher reactivity in ELISA than did the equivalent N-lung-I fractions, whereas materials eluting between Tubes 90–100 from the N-lung-I preparation showed considerably more reactivity than the equivalent C-lung-I fractions. Thus the antiserum used to monitor the eluted fractions reacted with both tumour and normal components. The antiserum as used in the complement-fixation assay appeared to be tumour-specific (Kelly & Levy, 1979), However, when used in the ELISA, it enabled us to identify the fractions from the Sephadex elutions that were most enriched for tumour specificity. Antiserum raised to the C-67 fraction demonstrated greater reactivity to C-lung-I in the ELISA than it did to N-lung-I, although it was not totally specific before adsorption. However, after passage of this antiserum over an immunoadsorbent column prepared with normal human serum, it demonstrated almost total specificity for the C-lung-1 antigen in ELISA (Fig. 3).

The apparent specificity of this system in ELISA led us to investigate the possibility that such an assay might be useful in detecting HTAA in the serum of patients with lung cancer. Preliminary studies with serum from normal individuals demonstrated that, though at a dilution of 1:10 serum could interfere with the assay, most normal sera at a 1:20 dilution did not significantly alter the results of the test (Fig. 4). A pool of normal sera was prepared from individual samples which did not significantly alter the development of ELISA at 1:20. This was used as a standard in running subsequent tests with serum from individuals with bronchogenic carcinomas, as well as additional serum samples from normal individuals. The results of a survey, done with both known normal sera (Fig. 5, Bar 1) and coded sera (Fig. 5) indicated that the serum from patients with early lung cancer was able to inhibit the development of ELISA at a dilution of 1:20, whilst sera from normal individuals did not. Some of the normal sera tested at 1:20 markedly enhanced the colour development in ELISA (Fig. 5, Bar 1). The reasons for this are not yet understood, although it has been suggested that high-serum immunoglobulin levels may account for it, since such results have been seen by others and accounted for in this way (Saunders, 1979). Further studies on the dilution of human serum in anti-C67 serum suggest the possibility of the presence of titratable HTAA in the serum of lung-cancer patients (Fig. 6). On surveying a number of human sera in mixture with anti-C67 and using N-lung I as the reacting antigen, it was found that none of the sera tested inhibited this reaction (Fig. 7). This observation diminishes the possibility that autoimmune reactions are responsible for these results. As one would expect, the degree of reactivity in this system between normal antigen and antibody to tumour-specific components is much less than that with tumour antigen (Fig. 3).

The finding that the serum from lung-cancer patients—when allowed to react with C-lung I antigen before reaction of the antigen with the rabbit antiserum (anti-C67)—could also effectively block the reaction, indicated that these sera might contain specific antibody as well as antigen (Fig. 8).

In summary, these studies have permitted us to develop a sensitive assay for HTAA in human bronchogenic carcinoma, and to demonstrate a component in the serum of patients with preoperative Stage I lung cancer which inhibits the reaction of a purified rabbit antiserum with fractionated HTAA of squamous-cell origin. This kind of inhibition has not been seen in any sera from normal individuals so far tested at a dilution of 1:20. Serum taken from a few patients with chronic obstructive lung disease and tested in this system in some cases showed marginal inhibitory activity (data not shown) but were never as inhibitory as the sera from the Stage I patients. The usefulness of these observations, either prognostically or diagnostically, can only be evaluated after further testing. The specificity of the reaction observed here in respect of bronchogenic carcinoma has not yet been ascertained, and the testing of sera from patients with other types of malignancy is part of a continuing study in this laboratory. The observation that patients with non-bronchogenic metastatic disease in the lung do not have inhibitory serum (Fig. 5) may indicate that there is some specificity in this test. However, other preliminary studies indicate that there may well be some cross-reactivity with some types of malignancy (work in progress). It is clear that a number of studies will have to be done, using sera from carefully staged patients, before these questions can be answered satisfactorily.

The authors thank the following people for their help in supplying human serum for this study: Dr H. Silver and Dr K. Karim, Cancer Control Agency of British Columbia, Vancouver, B.C., Canada; Dr P. Wright and Dr L. Hill, The Fred Hutchinson Cancer Research Center, Seattle, Washington, U.S.A.; and Dr W. Nelems, St Paul's Hospital, Vancouver, B.C., Canada. The authors also thank Dr Nelems for his support and enthusiasm during the early phases of this work, and Dr Ann Jackson for her help in preparing the alkaline phosphatase conjugates.

This work was supported by Grant No. 6048 from the National Cancer Institute of Canada.

### REFERENCES

BRAATZ, J. A., MCINTIRE, K. R., PRINCLER, G. L., KORTRIGHT, K. H. & HERBERMAN, R. B. (1978) Purification and characterization of a human lung tumor-associated antigen. *J. Natl Cancer Inst.*, **61**, 1035.

ENGVALL, E. & CARLSSON, H. E. (1976) Enzyme-linked immunosorbent assay (ELISA). In *First International Symposium on Immunoenzymatic Techniques*. Ed. C. Feldman. Amsterdam: North-Holland. p. 135.

KELLY, B. & LEVY, J. G. (1977) Evidence for a common tumour-associated antigen in extracts of human bronchogenic carcinoma. *Br. J. Cancer*, **35**, 828.

KELLY, B. & LEVY, J. G. (1979) Purification of a protein associated with human bronchogenic squamous-cell carcinoma. *Br. J. Cancer*, **39**, 224.

KELLY, B. S., LEVY, J. G. & SIKORA, L. (1979) The use of the enzyme-linked immunosorbent assay (ELISA) for the detection and quantification of specific antibody from cell cultures. *Immunology*, **36**, 989.

MOHR, J. A., NORDQUIST, R. E., RHOADES, E. R., COALSON, R. E. & COALSON, J. J. (1974) Alveolar cell carcinoma-like antigen and antibodies in patients with alveolar cell carcinoma and other cancers. *Cancer Res.*, **34**, 904.

MÖLLER, G. (1969) Immune responses by lymphocytes; their nature and regulation. In *Immunological Tolerance*. Eds. Landy & Braun. London: Academic Press. p. 215.

PORATH, J., AXEN, R. & ERNBACK, S. (1969) Chemical coupling of proteins to agarose. *Nature*, **215**, 1491.

SAUNDERS, G. C. (1979) The art of solid phase enzyme immunoassay including selected protocols. In *Immunoassays in the Clinical Laboratory*. Ed. R. M. Nakamura. New York: Alan Liss, Inc. p. 99.

SEGA, E., NATALI, P. G., RICCI, C., MINEO, C. T. & CITRO, J. (1974) Lung cancer tumour association antigen: Isolated by gel filtration and characterization by immunodiffusion. *Int. Res. Communications System*, **2**, 1278.

VÖLLER, A., BIDWELL, D. & BARTLETT, A. (1976) Microplate enzyme immunoassays for the immunodiagnosis of viral infections. In *Manual of Clinical Immunology*. Eds. Rose & Freedman. Am. Soc. Microbiol., Washington, D.C., p. 506.

WATSON, R. D., SMITH, A. G. & LEVY, J. G. (1975) The detection of immunodiffusion of tumour associated antigenic component in extracts of human bronchogenic carcinoma. *Br. J. Cancer*, **32**, 300.

YACHI, A., MATSURA, Y., CARPENTER, C. M. & HYDE, L. (1968) Immunochemical studies on human lung cancer antigen soluble in 50% saturated ammonium sulfate. *J. Natl Cancer Inst.*, **40**, 663.